# Leveraging IoT-Aware Technologies and AI Techniques for Real-Time Critical Healthcare Applications

**DOI:** 10.3390/s22197675

**Published:** 2022-10-10

**Authors:** Angela-Tafadzwa Shumba, Teodoro Montanaro, Ilaria Sergi, Luca Fachechi, Massimo De Vittorio, Luigi Patrono

**Affiliations:** 1 Department of Engineering for Innovation, University of Salento, 73100 Lecce, Italy; 2 Istituto Italiano di Tecnologia, Center for Biomolecular Nanotechnologies, Arnesano, 73010 Lecce, Italy

**Keywords:** internet of things, edge intelligence, healthcare and wellness, piezoelectric sensors, multi-sensor, anomaly detection

## Abstract

Personalised healthcare has seen significant improvements due to the introduction of health monitoring technologies that allow wearable devices to unintrusively monitor physiological parameters such as heart health, blood pressure, sleep patterns, and blood glucose levels, among others. Additionally, utilising advanced sensing technologies based on flexible and innovative biocompatible materials in wearable devices allows high accuracy and precision measurement of biological signals. Furthermore, applying real-time Machine Learning algorithms to highly accurate physiological parameters allows precise identification of unusual patterns in the data to provide health event predictions and warnings for timely intervention. However, in the predominantly adopted architectures, health event predictions based on Machine Learning are typically obtained by leveraging Cloud infrastructures characterised by shortcomings such as delayed response times and privacy issues. Fortunately, recent works highlight that a new paradigm based on Edge Computing technologies and on-device Artificial Intelligence significantly improve the latency and privacy issues. Applying this new paradigm to personalised healthcare architectures can significantly improve their efficiency and efficacy. Therefore, this paper reviews existing IoT healthcare architectures that utilise wearable devices and subsequently presents a scalable and modular system architecture to leverage emerging technologies to solve identified shortcomings. The defined architecture includes ultrathin, skin-compatible, flexible, high precision piezoelectric sensors, low-cost communication technologies, on-device intelligence, Edge Intelligence, and Edge Computing technologies. To provide development guidelines and define a consistent reference architecture for improved scalable wearable IoT-based critical healthcare architectures, this manuscript outlines the essential functional and non-functional requirements based on deductions from existing architectures and emerging technology trends. The presented system architecture can be applied to many scenarios, including ambient assisted living, where continuous surveillance and issuance of timely warnings can afford independence to the elderly and chronically ill. We conclude that the distribution and modularity of architecture layers, local AI-based elaboration, and data packaging consistency are the more essential functional requirements for critical healthcare application use cases. We also identify fast response time, utility, comfort, and low cost as the essential non-functional requirements for the defined system architecture.

## 1. Introduction

The Internet of Things (IoT) paradigm has rapidly gained popularity over the years resulting in billions of connected devices applicable to everyday scenarios in various industries [1]. Researchers and industry players alike have developed applications that leverage IoT-enabling technologies to develop intelligent environments such as smart cities [2,3,4], smart factories [5,6,7], and smart homes [8,9,10]. Consequently, the healthcare and wellness domain has also seen an increase in the use of wearable devices due to a growing demand for personalised healthcare, advances in the development of miniaturised flexible sensing technologies, and the proliferation of IoT technologies in modern society [11,12].

On the other hand, because of the technological advances applied to healthcare to increase the human lifespan, the number of elderly citizens in many developed countries around the world is increasing. The increased elderly population, as a result, puts a significant burden on existing healthcare systems and infrastructures since elderly citizens require constant care, assistance, and monitoring because of the numerous chronic illnesses and conditions related to ageing. In addition to the increasing number of elderly citizens, a general increase in the number of people suffering from chronic illnesses coupled with the worldwide shortage of healthcare workers also contributes to the burden on healthcare infrastructures [13,14,15,16]. Several governments have dedicated significant resources to developing innovative technological solutions to provide efficient, affordable, and non-invasive services to improve overall citizen quality of life. Therefore, several architectures based on IoT-enabling technologies and wearable devices have been designed and developed to improve the overall healthcare services offered to citizens and alleviate the burden on existing healthcare infrastructures [17,18].

Some of the developed architectures are based on wearable devices that involve the use of various combinations of physiological and environmental data collected by wearable sensors to diagnose illnesses and provide warnings and intervention solutions in some cases. In most cases, applying Machine Learning (ML) and Artificial Intelligence (AI) algorithms capable of inferring meaningful patterns from the collected data provides diagnoses and intervention solutions. However, in many existing frameworks, computationally expensive and Cloud-reliant methods and algorithms are employed to infer patterns and meaningful information from the collected data [19,20,21]. Therefore, for these Cloud-based frameworks to function, frequent Cloud access and data transmission from wearable devices to centrally located Cloud data centres are required, raising privacy and latency concerns. Challenges related to achieving secured transmission using Wide Area Network (WAN) communication technologies such as WiFi or 4G wireless technologies usually used to obtain Cloud access largely contribute to privacy concerns [22,23].

In contrast, the large distances between the data sources and Cloud data centres mainly contribute to latency concerns. Slow response times in Cloud-based architectures also arise because warning or intervention solutions originate from the same Cloud data centres located far away from the user [19,24,25]. This property, as a result, confines the application of these frameworks to application use cases where real-time or timely interventions are not functional requirements, thus limiting the range of possible healthcare services offered by the Cloud-based IoT frameworks [18,21,26,27,28]. In response to these concerns and limitations, the Edge and Fog Computing paradigms facilitated the realisation of IoT-enabled healthcare application frameworks offering better response time and privacy preservation [27,29].

AI techniques such as ML, Deep Learning (DL), Federated Learning (FL), or Continual Learning (CL) algorithms are also applied to Edge/Fog Computing infrastructures to allow for intelligent data processing at the network edge. Intelligent data processing at the network edge further reduces the application response time and improves the privacy offered to users [24,26,28,30]. Improved response time is particularly essential in time-critical applications, such as healthcare architectures, where quickly obtaining usable information from sensor data is extremely important, and delayed intervention may be fatal.

In addition to the computational considerations mentioned above, utilising advanced, high accuracy, and precision sensing technologies in the right application-specific combinations also improves IoT healthcare frameworks’ overall capabilities, accuracy, and robustness. To that end, cutting-edge research has been conducted in recent years to facilitate the creation of sensors capable of monitoring pertinent physiological signals with high accuracy and precision while utilising minimal power. Sensors made from bio-compatible materials easily attached to the skin and designed with comfortable form factors that allow them to cause limited to no intrusion to the user’s day-to-day activities are crucial in wearable-based healthcare frameworks. Additional information about such sensing technologies can be found in [11,31,32,33]. Adding multiple advanced sensors to one framework can provide valuable correlating data to make more meaningful and complex predictions. As a result, wearable-based IoT-aware healthcare frameworks based on multiple advanced sensors are more versatile, robust, and trustworthy. A reference for some healthcare application-specific sensor combinations is available in a survey published by Sabry et al. in [34]. Consequently, adopting miniature, flexible, skin-compatible sensor technology introduces the possibility of unobtrusively monitoring physiological parameters that are usually imperceptible using the typical, commercially available wearable devices [35]. Ultimately, this allows the definition, design, and development of IoT-based healthcare frameworks that facilitate the long-term surveillance of critical parameters in prevention, diagnosis, and rehabilitation.

Several frameworks based on Edge/Fog Computing paradigms have been successfully applied in the healthcare domain through telemedicine, e-health, and mobile health applications. Other application domains have, however, seen the addition of on-device intelligence improve response times and privacy preservation in IoT frameworks. This result means that in addition to AI algorithms applied to the framework’s Edge/Fog/Cloud nodes, sensing devices equipped with AI capabilities allow some on-device data analysis and, consequently, increase the amount of sensor data that can be exploited to perform the analysis, ultimately improving framework efficiency. Adding on-device intelligence can also limit data transmission between framework components, thus improving privacy preservation and device power efficiency. Typically, IoT frameworks rely on Bluetooth Low Energy (BLE), ZigBee, or other limited-range communication technologies to transmit raw sensor data to Edge/Fog nodes for processing. Therefore, introducing on-device data processing using ML and AI, regardless of complexity, could result in significant response time and power efficiency improvements by reducing the amount of data transmitted over wireless networks. However, this type of local on-device data processing based on AI is still in its infancy in healthcare domain applications. Therefore, it is necessary to define implementation guidelines, tools, and technical requirements for its adoption and integration with existing reference architectures [25].

Due to advances in the growing field of on-device AI, advances in sensing technologies, and the success of Edge/Fog/Cloud-based IoT frameworks, we propose that combining these technologies to develop healthcare domain frameworks can significantly contribute to the definition of reliable personalised healthcare architectures. In this paper, we, therefore, provide a detailed review of existing IoT-based architectures that utilise wearable devices for various healthcare applications. We also describe the requirements and reference architecture for a multi-layer IoT-aware system based on an advanced multi-sensor network leveraging Edge Computing technologies and on-device intelligence for critical or time-sensitive healthcare domain applications. The proposed architecture leverages advanced sensing technologies that allow the measurement of minute and accurate biosignals, low-cost communication technologies to facilitate the development of affordable wearable devices, Edge Computing in conjunction with Edge and on-device Intelligence technologies to facilitate secure real-time applications, and Cloud technologies to facilitate complex data analysis.

The main contributions of this paper are as follows:

- We provide a detailed analysis of the evolution of IoT-based architectural configurations applied to the healthcare domain.
- We define requirements for next-generation intelligent IoT-enabled personalised healthcare architectures. We define functional and non-functional requirements based on observations made from the existing literature and trends related to existing and emerging technologies while also considering the nature of the application scenarios.
- We define a detailed reference architecture configuration that combines high-precision sensing technologies, on-device intelligence, Edge Intelligence, and Cloud Intelligence. We also introduce technologies applied in the various components of the defined architecture to aid the successful implementation of the defined architecture.
- We present potential use cases and scenarios for which the proposed architecture can be adopted to guide researchers and interested parties.

The rest of the paper is organised as follows: Section 2 provides the review of existing IoT-based healthcare architecture configurations, Section 3 describes the requirements for the proposed IoT system architecture, Section 4 defines the proposed architecture, and Section 5 proposes potential application use cases that can benefit from adopting the proposed architecture. Finally, Section 6 gives conclusions and recommendations for future work.

## 2. State-of-the-Art

**Figure 1:**
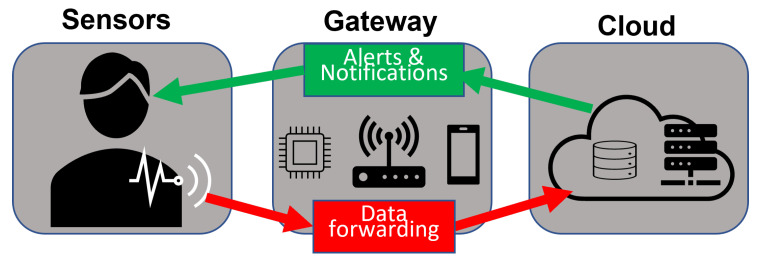
Typical Cloud-based architecture.

Many authors have presented various architectural configurations for healthcare domain IoT-aware systems based on wearable devices. These configurations can be classified into two main categories based on the approach used to acquire, store and, most importantly, process the collected sensor data. The discussion presented here is, therefore, divided into two main parts describing the two main architectural configuration approaches adopted in the literature. The first, a centralised architecture approach, involves the transmission of raw data directly from the sensing devices to the Cloud without the use of an intermediate layer, while the second, a decentralised architecture approach, involves the use of one or more intermediate layers that perform elaboration of data, provide temporary storage, or application-specific decisions between the sensors and the Cloud. Solutions within the first group usually leverage the Cloud to process data using either domain-specific non-AI algorithms or AI algorithms to obtain intervention decisions, diagnosis conclusions, or recommendations. They typically conform to the structure illustrated in Figure 1. In the first part of this section, we report the works that leverage the Cloud for storage, processing, and decision making and do not offload any of the computational tasks or offer user services through intermediate layers. While in the second part, we describe the works that involve the use of multi-layer architectures to support one or more of the following:

(a) data collection from wearable sensors,
(b) elaboration of data in Edge nodes,
(c) the temporary storage of data in Edge/Fog nodes,
(d) forwarding of information through gateways in the form of routers, switches, mobile phones, or specialised embedded systems, etc.,
(e) use of Artificial Intelligence for data analysis on Edge/Fog nodes,
(f) the exploitation of intelligence resources provided by a Cloud server.

The first architecture configuration falling under the first group was presented by Ahamed et al. in [36], who defined a generic architecture that combines IoT-aware wearable devices with Machine Learning and Cloud Computing techniques for the prediction of heart disease. In this architecture, the wearable devices transmit data directly to a Cloud platform containing data processing, storage, and visualisation facilities that can be accessed by the patient or medical practitioners from anywhere. Further, falling under this group is the work by Addante et al. [37] in which a system containing a forearm-worn wearable device that makes use of a combination of accelerometers and gyroscopes to monitor movement and EMG sensors to obtain muscle mass information for the diagnosis of Sarcopenia, an ageing-related muscular disorder, was defined. They also used BLE to transfer data between the measuring device and a mobile device hosting an application to interact with the measured data and function as a gateway to the Cloud database. Another framework where all forms of data processing are performed using Cloud infrastructure for the diagnosis and monitoring of chronic diseases with a focus on diabetes was developed by Abdali-Mohammadi et al. [38]. In their work, a combination of wearable and implantable sensors were used to collect patient physiological parameters, which were then directly transmitted to the Cloud using 3G/4G communication networks. The developed system also included the possibility of identifying emergencies and notifying nearby hospitals, allowing emergency care provision. Further, also falling within this group is the work defined in [39], which described a framework for monitoring, predicting, and diagnosing heart disease using a combination of IoT sensors and Cloud-implemented ML classification algorithms trained using data from existing repositories. Kumar and Gandhi [40] also defined a healthcare monitoring architecture that utilised data from IoT wearable devices. The collected data were directly stored and processed using Cloud-based techniques, namely Apache HBase [41] for data storage and Apache Mahout [42] for the prediction model. Similar to the works described above, several other works, such as the ones presented in [43,44,45], describe IoT-aware healthcare architectures in which raw data collected from IoT devices are directly forwarded to the Cloud through various gateways using diverse wireless network technologies. As seen from the discussion above, various technology alternatives were adopted to fit specific requirements or application scenarios and improve the efficiency and reliability of Cloud-based IoT infrastructures. However, Cloud-based architecture configurations are characterised by privacy and latency issues mainly because of the centralised Cloud server location and the network infrastructure used for communication and data transfer. Therefore, based on their demand for speed, accuracy, and reliability, time-sensitive real-time solutions cannot be achieved using this approach. Furthermore, since continuous sensing devices produce large amounts of data, architectures solely reliant on Cloud Computing resources to process and analyse all the data put a strain on the network and the sensing devices, which usually have limited power available. The second group of works discussed in this section attempts to address these shortcomings and facilitate robust solutions with improved service delivery. The distributed architectures discussed in this section still use the previously discussed sensor-gateway-cloud 3-layer template; however, the gateway is realised through Edge or Fog Computing paradigms. The intermediate layer(s) perform varying levels of data elaboration, analysis, and application service delivery offering varied scales of improved efficiency, scalability, reliability, latency, privacy, and security. Some other works described in this section also implement AI algorithms at the framework edge, i.e., on nodes located in close proximity to the sensors, further improving the abovementioned parameters.

Gia et al. [46] presented an IoT for healthcare architecture for fall detection and monitoring heart rate variability that exploits Fog Computing technologies to improve the previously mentioned latency and security concerns and the lifetime of the sensing devices. The defined architecture utilises wearable electrocardiogram (ECG), motion, and body temperature sensors with environmental room temperature and humidity sensors to collect data. The sensing devices forward data through a low-cost RF interface to gateways that offer Fog services, including short-term data storage, data filtering, data processing, and generating near-real-time push notifications to inform the user and authorised health professionals of any concerning events or abnormalities. In this application, historical data can be accessed through the Cloud. However, the short-term storage available on the Fog node is also accessible through a local network, thus providing service reliability in the event of a network interruption. Similarly, Hajvali et al. [47] presented a generic software architecture for real-time IoT healthcare systems that contains a partitioned two-level Fog Computing layer. The two layers distribute the services available at the edge to reduce the number of tasks performed by one device, which results in a faster response time. The architecture also includes a smartphone that acts as a gateway between the sensor devices and the Fog layer and, in addition, hosts an application with a GUI that facilitates the accessing of alerts from the Fog node and local data management and visualisation. The architecture also includes a Cloud component responsible for further data processing and storage. The Cloud component also provides users and interested parties access to the database and user interfaces. The authors of this work focused their attention on user mobility; therefore, they emphasise the description of a software set-up that allows a user continuous access to a Fog node even if their physical location changes.

However, as established in previous sections, in addition to simply adding specific data processing algorithms to the Fog/Edge layers, AI algorithms can also contribute to the achievement of fast response times and improvement of the accuracy of the decisions made by IoT-aware health monitoring systems. As a result, several architectures that include AI algorithms applied to the Fog layer have been developed. For instance, the authors of [48] developed an architecture based on RF communication technology that implements temperature, ECG, blood pressure, and blood oxygen measurement with the aid of Fog and Cloud Computing technologies for remote monitoring of pregnant women. In this scenario, the Fog node, in the form of a Raspberry Pi, is responsible for user authentication, feature extraction, classification of collected data using a Bayesian Belief Network (BBN), and issuing alerts to health practitioners if a critical event is detected. On the other hand, the authors of [49] developed an integrated environment that incorporates Deep Learning (DL) algorithms in Fog nodes for an application for coronary disease monitoring and diagnosis. This application has two types of Fog nodes, namely, broker and worker nodes, to distribute the computational tasks. Ribiero et al. [50] also describe an architecture that leverages AI algorithms and advanced mathematical models in both the Fog and Cloud nodes. The proposed architecture accurately performs localised fall detection and classification; however, it does not leverage wearable sensor technology to measure parameters related to fall events.

In a nutshell, IoT-aware health monitoring system architectures fall into two main groups: centralised and decentralised architectures. Centralised architectures were the first to be adopted for periodical monitoring and continuous monitoring frameworks where collected sensor data are forwarded directly to the Cloud for processing. On the other hand, decentralised architectures have distributed data processing and service delivery capabilities in the Edge, Fog, and Cloud nodes per application requirements. In decentralised architectures, the prevailing trend has, until recently, seen AI and ML algorithms applied to Cloud and Edge/Fog nodes to perform data processing and analysis. However, recently, an additional sub-class of decentralised architectures where AI algorithms are implemented directly on wearable devices has emerged. An example of this type of architecture is described by Arikumar et al. [29], who proposed a Person Movement Identification (PMI) framework with AI algorithms for automatic feature extraction on the wearable device and classification in Edge and Cloud nodes. The architecture they defined implements a distributed continuous learning approach that enables on-device processing of data collected from multiple sensors and accounts for differences in user-related features. This emerging architecture allows the realisation of fast, accurate, scalable, and reliable health monitoring architectures suitable for personalised applications. However, as illustrated in Table 1, none of the articles available in the literature provides user services after on-device data processing.

Based on the observations made from the conducted research, obtaining some useful information and generating alerts or alarms after the elaboration on-device would significantly improve the response time and, consequently, the efficiency and service delivery offered by the IoT framework. Additionally, to aid the framework design and guide future research in this field, we define requirements and outline the various components of a reference architecture that incorporates on-device intelligence and early service delivery in IoT-based healthcare frameworks.

## 3. System Requirements

This section, based on the analysed literature and inspired by the solutions proposed in the papers discussed in the previous section, outlines the requirements we defined for a comprehensive and versatile multi-level architecture. The presented architecture can be adopted by researchers interested in utilising an approach that combines IoT, AI, Edge/Fog Computing and multiple advanced sensing technologies to their healthcare domain application solutions. In addition, the presented architecture also allows the inclusion of Cloud Computing technologies since they are still an integral part of IoT architectures, as demonstrated by the trends in the literature.

The first consideration that guided our architecture design is the most commonly used structure in the analysed works. As discussed in previous sections, most of the architectures defined in the literature conform to a 3-layer structure, like the one illustrated in Figure 1, whereby data processing and storage of sensor data are centralised in the Cloud. In some cases, however, the defined architecture includes Edge Computing or Fog Computing layers to overcome the shortcomings of the centralised architecture. It has also been established that the response time, capabilities, efficacy, and fidelity of real-time IoT-aware architectures can be further improved by introducing Artificial Intelligence to the sensing devices. The quality of service offered by the architecture can also be enhanced by providing some insights related to the application scenario after the initial on-device elaboration. Therefore, based on these observations, an improved solution could be a modular system that distributes duties among different modules and utilises sensing devices with AI capabilities. In this case, end-user sensing devices can be used to collect the data from each sensor, perform local elaboration (e.g., filtration, simple analysis, or anomaly detection), and then forward results to a superior Edge/Fog node where further elaboration and analysis can be performed. In this way, user privacy can be preserved by avoiding the direct transmission of raw data collected from sensor devices. In addition, the powerful Edge/Fog node can also contribute to the preservation of privacy by further elaborating the initial results and only sharing inferred results to the last element of the architecture in the Cloud. It is also essential for the system to have the ability to add different, new sensors without affecting the other layers. Based on these considerations, the following list summarises the functional and non-functional requirements we have collated to guide the research community working within this context in the design of a comprehensive architecture.

### 3.1. Functional Requirements

The functional requirements listed below are essential to achieve the desired functionality.

**FR1:**  Distribution of the duties among different modules/layers.
**FR2:**  Modularity—Independence of each layer to guarantee the possibility of changing each layer with, for instance, a new version of the implementation software at runtime without affecting other layers.
**FR3:**  A first local layer to perform some elaboration of the collected raw sensor data and package the results in a standard packet format to be transmitted to the next layer.
**FR4:**  A second local layer that is able to receive data from the previous layer in the same standard packet format used by the first layer.
**FR5:**  The same second layer should support multiple packet formats.
**FR6:**  The same second layer should locally store the data in a buffer to be robust to connection problems.
**FR7:**  Bidirectional communication between the first and second layers.
**FR8:**  The same second layer should be able to forward the data, in a standard format, after some further elaboration to a Cloud server.
**FR9:**  Cloud server should be able to receive data in a standard format.

### 3.2. Non-Functional Requirements

The requirements listed in the current sub-section do not affect the technical functionality. However, they are related to the architecture’s performance, accuracy, acceptability, utility, and adaptability to application scenarios.

**NFR1:**  Response time and accuracy—considering the nature of the application scenario, slow response time or inaccurate outputs could have fatal consequences; therefore, the system must guarantee accurate output and near-real-time response times.
**NFR2:**  Usability—the system should avail user-friendly interfaces to facilitate easy exploitation of exposed services.
**NFR3:**  Comfort—considering the nature of the scenario, i.e., continuous healthcare monitoring, the system should guarantee the use of highly comfortable monitoring devices not only to ensure user acceptance but to ensure they are willingly accepted.
**NFR4:**  Performance and Interaction—the system should guarantee reactivity to all user requests and interactions.
**NFR5:**  Reliability, Availability, and Maintainability—the system should be able to work and expose services without failures.
**NFR6:**  Scalability—considering the proposed system’s mobile nature, it would support the possibility of increasing the number of users and, therefore, sensors in more locations.
**NFR7:**  Low-Cost—considering the application scenario, users and healthcare officials would prefer low-cost architectures to provide affordable healthcare infrastructures.
**NFR8:**  Low-Power—considering the presence of battery-powered mobile healthcare monitoring devices, one of the most important non-functional requirements regards the provision of energy savings.
**NFR9:**  Security—the system should guarantee that all the manipulated data and all system components are protected against malicious attacks or unauthorised access.

## 4. System Architecture

**Figure 2:**
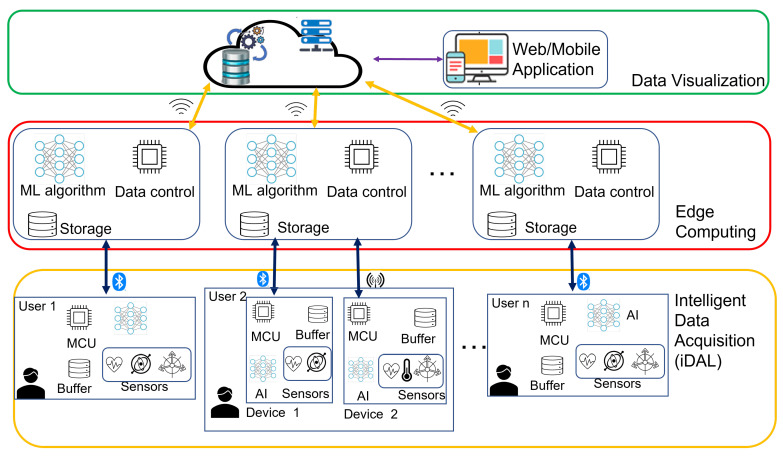
Proposed system architecture.

Based on the analysis reported in the literature review and the consequent observations and requirements defined previously, this section presents the architecture for a real-time IoT-aware healthcare system that incorporates advanced multi-sensing technologies, Edge-based and on-device AI components. The proposed architecture contains three main layers, namely, (i) Intelligent Data Acquisition Layer (iDAL), (ii) Edge Computing Layer, and (iii) Data Visualisation Layer, as illustrated in Figure 2. One of the critical features of the proposed architecture is the modularity and distribution of duties among components to facilitate easy upgrade and fulfil functional requirements, FR1 and FR2.

This section highlights the interactions between the different layers and modules of the proposed architecture and introduces their function and possible composition.

### 4.1. Intelligent Data Acquisition Layer (iDAL)

**Figure 3:**
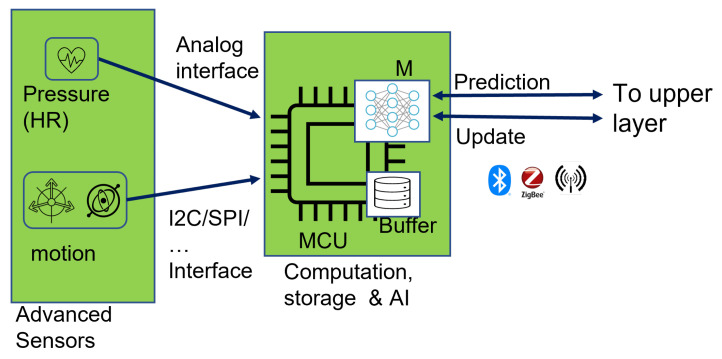
Intelligent Data Acquisition Layer.

The first layer is the Intelligent Data Acquisition layer (Figure 3), with three major components: (a) advanced sensors, (b) a computational and storage unit, and (c) an Artificial Intelligence module.

#### 4.1.1. Advanced Sensors

Various sensing technologies can be used to collect data relevant to the specific health-related parameters of interest applicable to the use case to which the architecture will be applied. For example, for a cardiac-related AAL application, a combination of skin-compatible piezoelectric or piezoresistive sensors defined in [31,32,33,52,53] in conjunction with motion, temperature, or positioning sensors, which add contextual information to the physiological measurements, can be used in this layer. The usage of flexible sensors can fulfil NFR3 and facilitate the development of comfortable and reliable wearable devices suitable for long-term surveillance of critical parameters in prevention, diagnosis, and rehabilitation use cases in the healthcare domain. Adopting advanced miniature flexible skin-compatible sensor technologies also introduces the possibility of accurately monitoring tiny physiological parameters that are usually imperceptible using the typical commercial, wearable devices [35], thus increasing the chances of fulfilling NFR1. Incorporating multiple sensors also allows the system to gather valuable correlating data that can be used to make more complex and meaningful deductions, thus making wearable-based IoT-aware healthcare frameworks more robust and trustworthy.

#### 4.1.2. Computation and Data Processing

The second part of the iDAL is the computational unit responsible for controlling the sampling and acquisition of sensor data. To be considered for this function are Commercial-off-The-Shelf (COTS) low-power and low-cost microcontroller units that can support the interfaces and data transfer protocols implemented by the selected sensors, such as analogue inputs, SPI, I2C, etc. Field-Programmable Gate Arrays (FPGAs) or Application-Specific Integrated Circuits (ASICs) can also be considered to achieve higher speed, flexibility, and exclusive control over the functionality, size, or device form factor; however, this would significantly increase the overall cost of development, development time, and, consequently, production.

#### 4.1.3. Artificial Intelligence Module

In addition to the primary signal processing techniques implemented to perform initial signal conditioning and processing, the attached microcontroller unit (MCU) is equipped with specific AI algorithms. The AI algorithms can perform data analysis to facilitate local decision-making by performing functions such as anomaly detection, high-level feature extraction, classification of the measured data, etc., to achieve real-time response and forwarding of processed data to upper layers and, thus, privacy preservation.

Some considerations also need to be made when selecting the Edge Intelligence technologies and, consequently, the level of data processing performed directly on the end devices through Artificial Intelligence. Some of the factors that govern the choice of AI algorithm used, the extent of data analysis, and the eventual output produced by the algorithm include device-related factors such as (a) the power consumption and available computational and storage capacity, (b) algorithm-based factors, such as complexity, computational, and storage requirements or results accuracy, and (c) application specific and operational factors, such as privacy concerns, latency requirements, etc. Several types of AI algorithms can be considered for this stage. For instance, the data-driven techniques for anomaly detection algorithms described in [28,54] could be applied. In cases where multiple sensors are all attached to the same computing/wearable device, instead of transmitting all the raw data from multiple sensors, the ML algorithms can be used to automatically extract the pertinent features from the combined sensor data, thus significantly prolonging the lifetime of the constrained battery-powered devices. Reducing the amount of data to be transmitted, in turn, reduces the required transmission time and, ultimately, the power consumption since communication is usually responsible for most of the device power cost of an IoT system. Pre-trained models that can be deployed to perform on-device inference may be used for simple anomaly detection, whereby the results can then be used to issue warnings if any unusual behaviour is observed or propose a course of action based on the algorithm predictions. In such cases, lightweight algorithms are worth considering based on the resource budget available in the MCU. Alternatively, a predetermined fraction of a large partitioned Neural Network can be implemented to perform partial on-device data elaboration. In this way, only intermediate results are forwarded to higher architecture levels for further elaboration, thus ensuring the preservation of privacy by avoiding the transmission of raw data as required by NFR3. As mentioned earlier, the amount of data to be transferred over a network is proportional to the overall device power consumption and the required bandwidth. Therefore, forwarding intermediate results reduces the bandwidth and power consumption cost, thereby enabling the fulfilment of NFR8. Based on these considerations, further investigation into the implementation and selection of the extent of on-device intelligence can be aided by consulting the architectures described in [30,55]. Sabry et al. [34] also highlight potential issues related to on-device intelligence in healthcare applications and provide some considerations to aid algorithm selection.

Various software tools, libraries, and frameworks have also been developed to facilitate the deployment of AI algorithms on constrained devices. For instance, TensorFlow lite for MCUs (TFLM) [56], an open-source library, can be used to support the deployment of Neural Networks (NNs) on a wide range of MCUs and Digital Signal Processors (DSPs). It has been tested on several Cortex-M series MCUs and can be used to deploy static algorithms trained using TensorFlow [57] to perform on-device inference. STMicroelectronics [58] also developed X-Cube-AI; a software tool that allows the generation and optimisation of AI algorithms developed using the typical ML and AI frameworks such as TensorFlow [57], Keras [59], or PyTorch [60] for deployment on the STM32 family MCUs. In addition, Edge Impulse [61] is a Cloud-based tool that allows the development of both NN and non-NN models for various embedded platforms such as MCUs or mobile phones. This tool allows the collection of sensor data directly from supported devices to train ML models, thus enabling fast prototyping of on-device ML architectures. Another available tool is NanoEdge AI Studio [62], which supports both learning and inference inside the MCU. This tool allows the automatic selection of ML libraries best fitting the provided data, making this tool suitable for developers with little or no AI or ML experience also. This tool contains libraries for the development of anomaly detection algorithms, one-class classification, multiple-class classification, or regression algorithms. The available tools can be selected based on available hardware and the developer’s expertise. Other available tools that can be leveraged for the deployment of the selected ML algorithm can be found in [34,63].

Finally, after the elaboration and analysis of data performed by the AI module in the iDAL layer is completed, the results, inferences, or generated alarms are packaged and forwarded to the upper layers for further processing or management. Figure 3 illustrates a graphical summary of an example implementation of this layer consisting of two sensor modules.

### 4.2. Edge Computing Layer (ECL)

The second layer defined to fulfil FR4 is the Edge Computing Layer, which is primarily responsible for receiving data from the iDAL and providing a gateway to the upper layer. The same layer is also responsible for handling and managing communication with devices that may be equipped with different communication protocols and performing further data analysis. This layer is capable of bidirectional communication with both the lower layer and the upper layer to:

(a) receive data from the iDAL via standard low-power communication protocols such as BLE
(b) send updates to the iDAL
(c) forward data to the upper layer
(d) receive updates or notifications from the upper layer

Based on the AI architecture selected in the previous levels, an AI algorithm can be deployed in this layer in its entirety or as a fraction of a partitioned model. The choice of this model is based on the resources available for computation, storage, or power. The ECL should support various communication protocols since it could be responsible for serving multiple iDAL nodes with diverse protocol requirements. This layer can also be used to contact authorised caregivers, relatives, or health personnel in the event of detected distress or undesired events.

### 4.3. Data Visualisation Layer

Finally, the third layer is the Data Visualisation layer, which interacts with the storage facilities and facilitates the provision of user interaction services. Authorised users can view available data such as warnings generated by local devices and customised views of historical events through a Web Dashboard exposed by this layer. Healthcare professionals can also use the dashboard to provide recommendations that users can receive through the available communication channels. This layer is also responsible for advanced data analysis facilitated by the Cloud infrastructure. In this case, a more traditional AI algorithm can be considered to facilitate the analysis of historical data. This layer also exposes REST APIs to allow interaction with lower layers and contains a database to store the received data and network configurations.

## 5. Discussion

As already discussed, in the present paper, we defined an architecture extracted from and inspired by the analysed literature to provide a general and modular architecture that researchers or practitioners in the healthcare domain can take into consideration and exploit in their future works and solutions. Therefore, this section presents examples of the possible scenarios that the designed architecture can serve through interesting use cases inspired by real situations and case studies presented in the literature.

The first scenario to which our architecture can be applied is the continuous monitoring of citizens with chronic heart conditions. Considering the grave implications of heart malfunction to the human body, patients with chronic heart conditions, especially the elderly population, cannot maintain a normal autonomous lifestyle. As a result, they require constant surveillance and must frequently visit hospitals for check-ups. To improve their autonomy and, subsequently, quality of life, the proposed architecture can be applied to perform continuous surveillance on parameters related to heart health. In this application scenario, flexible piezoelectric sensors placed on different body parts (chest, ankle, or wrist) can accurately monitor cardiac function because of their capability to detect minute signal changes. In addition to their accuracy, each sensor, if placed correctly, can also be used to simultaneously monitor multiple parameters, such as heart sounds, blood pressure, and heart rate. Such a framework eliminates periodic blood pressure (BP) checks using the typical cumbersome cuffs by providing continuous BP insights. The frequency of hospital visits to perform periodic heart health check-ups can also be significantly reduced. In addition, an anomaly detection model can be deployed on the device to facilitate the fusion of the sensor data and extraction of parameters. The extracted parameters are transferred to the Edge Computing Layer (ECL), which performs further classification on the detected anomalies. The resulting ECL classifications can then be used to provide recommendations to the user and selected concerned parties. The implementation of Edge and on-device intelligence allows the system to provide real-time notifications, alarms, and recommendations, thus affording users more independence and autonomy.

Another application scenario that could benefit from this architecture is diagnosing, monitoring, and managing patients with neurodegenerative diseases. An Edge AI algorithm can be applied to perform feature extraction and anonymise data collected from a predetermined combination of motion and motor function sensors. The collected information can be used to provide real-time activity recommendations to assist patient rehabilitation or facilitate the provision of real-time feedback while patients are performing their recommended rehabilitation exercises. Caregivers can also remotely monitor patient progress and provide feedback, when necessary, through the web services exposed by the system.

Furthermore, on a larger scale, the architecture can be implemented in nursing homes or communities, especially those populated by the elderly, towards the realisation of self-sustainable smart cities. In this scenario, citizens are equipped with iDAL nodes to measure pertinent physiological parameters. ECL nodes can then be placed in strategic locations within the community to ensure complete coverage. In this case, implementing the Edge Intelligence of the individual iDAL nodes assures the user that their data remain private.

Finally, the presented architecture provides a solution that can be applied to improve the majority of the works discussed in the literature analysis and aid the design of new IoT-based healthcare frameworks for novel application use cases. As mentioned in earlier sections, adding on-device data processing, no matter how limited, can significantly improve various performance parameters of IoT infrastructures for healthcare applications. Therefore, using the results from the on-device analysis could significantly improve the efficacy and efficiency of the defined architectures by signalling any anomalies or concerns as early as possible. For instance, the fall detection architecture defined in [29] that uses federated learning and on-device feature extraction could allow the wearable device to produce an alarm to prevent falls. Additionally, adding Edge and on-device AI and including alarms generated by the pregnancy monitoring system defined in [48] would render the system applicable to critical pregnancies, where the mothers require close monitoring. Any detected abnormalities would be immediately signalled even when the user is far away from a health facility.

In the remainder of this section, we describe a solution we adopted to implement the early notification and anomaly detection functionality we propose for the iDAL layer and how it can interact with the upper layers.

### 5.1. iDAL On-Device Intelligence Implementation

The bottom-most layer of the proposed architecture hosts intelligent sensors capable of data processing and, if required by the application, providing a user service such as notifications and warnings. The state-of-the-art analysis we performed revealed the need for implementing data processing methods to increase the speed at which the system provides user feedback to improve the overall service delivery offered by IoT healthcare frameworks. One of the possible methods of providing user feedback, as has been discussed in earlier sections, is the implementation of anomaly detection algorithms on the sensing device to (a) provide onboard data processing, (b) reduce the amount of the data transmitted to the upper layer, and ultimately, (c) provide quick preliminary user feedback. Therefore, the first experimental work contributing towards implementing the architecture proposed in this work involves implementing and evaluating a prototype of the iDAL layer from our architecture with specific emphasis on the AI section.

This section describes the implementation of an on-device anomaly detection algorithm developed to fulfil FR3 of the proposed architecture. The requirement calls for *“a local layer to perform some elaboration of the collected raw sensor data and package the results in a standard packet format to be transmitted to the next layer"*.

#### 5.1.1. AI Algorithm

**Figure 4:**
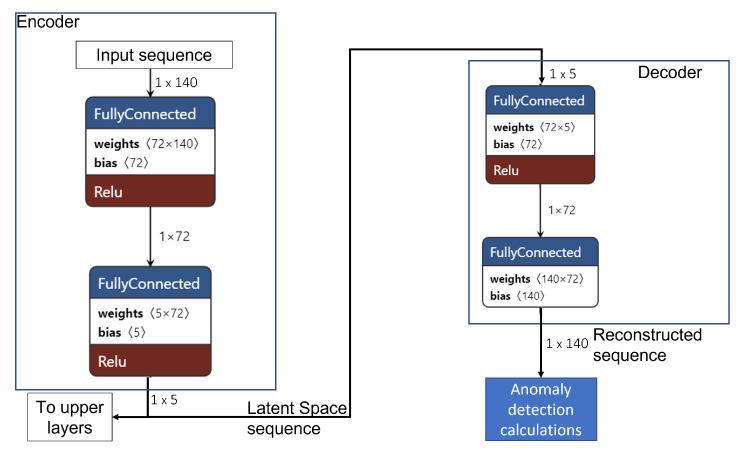
Autoencoder with separated Encoder and Decoder.

**Figure 5:**
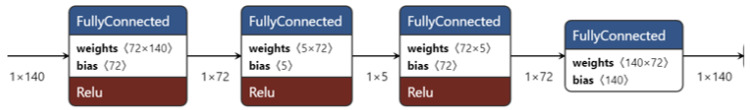
Simple Autoencoder structure.

Using an ECG monitoring scenario, we designed and deployed the simple pre-trained anomaly detection algorithm with the structure illustrated in Figure 4 on an MCU. The deployed algorithm is composed of an encoder to compress an input sequence into a smaller dimension and a decoder that attempts to reconstruct the input sequence from the compressed data. For this work, we modified the algorithm we evaluated in [64] (Figure 5) by defining and deploying the encoder and decoder as separate models, thus allowing us to access the encoder output in addition to the anomaly prediction during inference. In this way, the encoder output can be transmitted to an upper layer device containing a copy of the decoder to perform the reconstruction, resulting in a reduction in the amount of data to be transmitted by the sensor device over the implemented wireless communication channel. In addition, the inference results can also be made available to the user with a significantly reduced latency. In the testing section, we verify the possibility of implementing the proposed on-device intelligence method to achieve the expected reduced latency and amount of transmitted data.

#### 5.1.2. Test Set-Up

**Figure 6:**
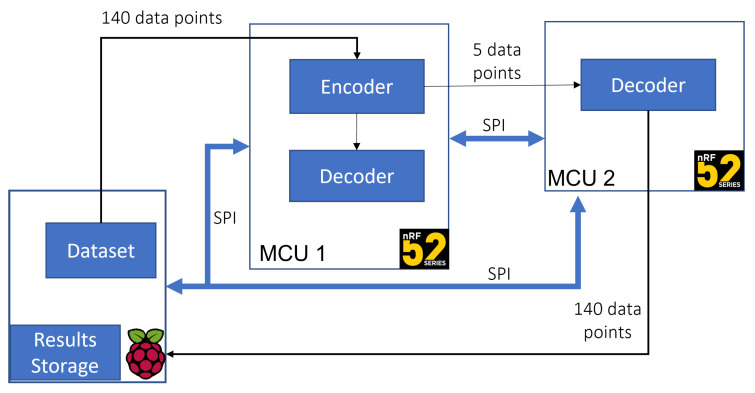
Test set-up.

As mentioned earlier, we adopted an ECG monitoring use case; therefore, the first step in the experimental procedure was training an autoencoder in TensorFlow [57] using the publicly available ECG5000 dataset [65]. To perform our tests, we adapted the dataset, which contains 5000 heartbeat samples obtained from monitoring a patient with severe congestive heart failure over 20 h. The original dataset samples are annotated with labels 1–5, where 1 represents a normal heartbeat, and the other labels, 2–5, represent different classes of abnormal rhythms. In the first phase, we focused on the normal samples to determine the autoencoder sample reconstruction ability. Therefore, from the 2919 normal samples, i.e., the samples annotated with label 1, we used 70% to train the model and reserved 30% for validation and testing. In exact numbers, the splitting produced 2043 randomly selected samples for training, and of the remaining 876 samples, the first 438 were used for validation, with the last 438 reserved for testing. The rest of the dataset samples from classes 2–5 were combined and annotated with label 0 to represent abnormal heartbeats to test the algorithm’s anomaly detection. We used the Google Colaboratory [66], an online notebook platform that allows browser execution of AI and ML algorithms, to design, validate, and test the algorithm. After training, validation, and testing in Google Colab, we converted the TensorFlow model into C byte arrays that we loaded onto a bare metal MCU to perform inference using the TensorFlow Lite for microcontrollers [56] and for an interpreter [64]. To verify the functionality, i.e., the reconstruction capability of the separated autoencoder, we developed the set-up illustrated in Figure 6. In the defined set-up:

1. The Raspberry Pi represents a sensor device that collects physiological signals in the test scenario.
2. The first nRF52 device represents the on-device intelligence computing module connected to the sensor module on which the complete autoencoder components, i.e., both the encoder and the decoder, are deployed.
3. The second MCU represents the Edge computing layer hosting the separate decoder model.

The inference results from both MCUs are transferred back to the Raspberry PI and compared to the expected results. In this test scenario, we used an SPI bus to transfer data between the on-device intelligence computer and the Edge Computing device to allow fast prototyping and high-speed data transmission. However, BLE, ZigBee, or similar wireless technologies can be used to link between the Edge computing device and the sensor module during the implementation phase.

#### 5.1.3. Results Discussion

**Figure 7:**
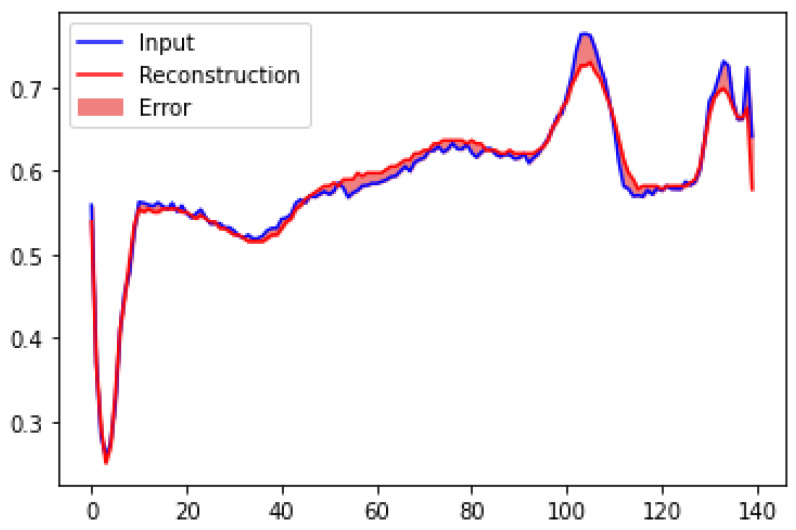
Original input vs. reconstructed decoder output.

One of the tests we performed was comparing the original dataset and the reconstruction obtained from the stand-alone decoder deployed on the Edge computing device. Figure 7 illustrates the reconstruction error between an ECG sequence from the original dataset and the reconstruction from the deployed decoder. We calculated an average reconstruction accuracy of 99.947% using the mean squared error of the difference between the original input sequences and all the reconstructed sequences from 438 test samples.

The second set of tests we performed was to determine the time required from the moment data are available for processing to the moment usable data for user consumption are made available. In this scenario, we define usable information as the prediction classifying a heartbeat as anomalous or normal. Our tests revealed a latency reduction of 3 ms obtained by considering two test scenarios:

- when available data samples are transferred over BLE to the Edge computing device when no data processing is performed on-device, and
- when the on-device anomaly detection algorithm is implemented on the sensor module using the configuration defined in Figure 3.

**Figure 8:**
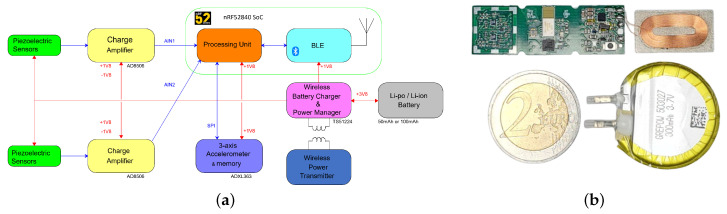
Custom BLE−enabled sensing module. (**a**) Block diagram, (**b**) prototype device to scale.

To obtain the time required to transmit data between the sensor module and the Edge computing device, we performed throughput tests using the prototype of a custom BLE-enabled sensing device pictured in Figure 8 connected to biocompatible piezoelectric sensors. The throughput test revealed a maximum reliable data rate of 16 kb/s for data transmission with no packet loss. Considering the ECG anomaly detection use case and the data formatting and segmentation parameters used to compile the ECG5000 dataset we used; the anomaly detection algorithm requires 140 data points as input. Therefore, based on this constraint, we obtained the following results from the two scenarios.

- Scenario 1: Based on the throughput tests, and, therefore, a 16 kb/s data rate, transmitting 140 samples from the sensor over BLE would require a minimum of 3.5 ms given a 2-byte digital representation with no additional data overhead. In addition to the transmission time, the total time required to obtain a usable result also includes the processing time required by the Edge computing device to perform inference and anomaly detection. Our tests revealed a minimum average processing time of approximately 0.48 ms to obtain an anomaly prediction. We measured the required inference time by counting the number of CPU cycles used by the MCU from the moment all the data samples are ready for elaboration to the moment the anomaly prediction result is available. Therefore, the total time required to obtain the first usable result and provide feedback to the user is 3.548 ms.
- Scenario 2: In this scenario, we consider the anomaly detection algorithm on the sensor module configured as illustrated in Figure 3. Transmitting the same data considered in the first scenario via SPI to perform on-device anomaly detection requires 0.028 ms with a 1 MHz SPI data rate. With the 0.48 additional milliseconds required for inference, the total time required to obtain a result that can provide valuable information to the user in this scenario is 0.508 ms.

According to the test results, implementing the on-device intelligence method described in this section successfully reduced latency (in this case, in reference to the time required to produce a usable inference result) by 3 ms. As mentioned before, the anomaly detection algorithm requires an input with 140 data points. However, implementing the autoencoder configuration described in this section allows the sensor module to send only five data points obtained from the encoder output to the Edge computing layer for further processing and record keeping. This result signifies a reduction in data that must be transferred over BLE by a factor of 28, from the initial 140 data points per sample to 5 data points per sample. Further data analysis can be performed using classification or other specific mathematical algorithms based on the reconstructed data obtained from the copy of the decoder deployed on the Edge Computing device. A comparison of the minimum timing and data requirements extracted from the two scenarios described above is summarised in Table 2.

## 6. Conclusions

The need for personalised and home-based healthcare architectures has been increasing over the years, driven by factors, including the growing percentage of elderly citizens, the global shortage of healthcare workers, and the need for overall improved healthcare services. In a bid to provide a solution, various researchers provide several technological infrastructures, systems, and frameworks based on technological innovations. However, most of the frameworks utilising IoT technologies developed to date are predominantly based on Cloud infrastructures characterised by problematic issues, such as privacy and latency, which are undesirable for interactive and critical healthcare applications.

In order to contribute to the resolution of these issues, this work presented a modular IoT-aware system architecture that can be applied to numerous application scenarios in the healthcare domain.

The presented architecture encourages the amalgamation of advanced sensing technologies, low-power and low-cost IoT enabling technologies, and emergent AI techniques to develop modular, reliable, and scalable critical healthcare infrastructures. In addition, the modular nature of the architecture permits its suitability for a wide range of use cases since it can be configured based on application requirements, as demonstrated in the discussion. We also discussed and demonstrated the benefits of implementing on-device intelligence from a latency and communication efficiency point of view. The added functionality of providing user alarms or notifications immediately after on-device AI-based data processing has the potential to revolutionise IoT-based healthcare infrastructures.

Further developments can improve the reliability and performance of the system, such as including blockchain technologies to increase scalability while enhancing security to maintain the desired preservation of privacy. 

## Figures and Tables

**Table 1:** Comparative analysis: Existing architectures.

Source	Cloud	User Service	Edge/Fog	User Service	AI	On-Device	User Service	Application
[36]	✓	Data access (GUI)	x	-	Cloud	-	-	Cardiovascular disease
[37]	✓	Data access (Web/Mobile App)	x	-	x	-	-	Sarcopenia
[38]	✓	Patient alerts	x	-	Cloud	-	-	Diabetes diagnosis
[39]	✓	Test Report	x	-	Cloud	-	-	Heart disease prediction
[40]	✓	Medical alerts (doctors)	x	-	Cloud	-	-	Heart disease prediction
		Data access (Web App)						
[43]	✓	Data access (Web App)	x	-	Cloud	-	-	Heart disease prediction
[44]	✓	Data access (GUI)	-	-	Cloud	-	-	Multiple disease prediction
		Alerts						
[45]	✓	-	x	-	Cloud	-	-	Diabetes prediction
[51]	✓	Data access (Web App)	-	-	x	✓PPG HR estimation	-	Elderly citizen health monitoring
[46]	✓	Data access (Web App)	✓	Push notifications Local host GUI	x	-	-	Human fall detection Heart rate variability
[47]	✓	Data access (Web App)	✓	Alerts Local Host GUI	x	-	-	Disease monitoring and prediction
[48]	✓	Authenticated data access	✓	Alerts	Cloud	-	-	Pregnancy e-health
[49]	✓	Data access	✓	Data access	Cloud & Edge	-	-	Heart disease monitoring
[50]	✓	-	✓	Alerts	Cloud & Edge			Human fall classification
[29]	✓	-	✓	-	Cloud & Edge & Device	✓Feature Extraction	-	PMI

**Table 2:** Tabulated summary of results discussion.

	No On-Device Intelligence	With On-Device Intelligence
Data transmission	3.500 ms	0.028 ms
Inference	0.480 ms	0.480 ms
Total required	3.548 ms	0.508 ms
Number of data points to upper layers	140	5

## Data Availability

Not applicable.

## Abbreviations

The following abbreviations are used in this manuscript:

IoT	Internet of Things
BP	Blood Pressure
FL	Federated Learning
DL	Deep Learning
ML	Machine Learning
AI	Artificial Intelligence
iDAL	Intelligent Data Acquisition Layer
ECL	Edge Computing Layer
API	Application Programming Interface
EMG	Electromyography
BLE	Bluetooth Low Energy
MCU	Microcontroller Unit
FPGA	Field Programmable Gate Array
ASIC	Application Specific Integrated Circuit
COTS	Commercial Off The Shelf
SPI	Serial Peripheral Interface
NFC	Near Field Communication
TFLM	TensorFlow Lite for Microcontrollers
DSP	Digital Signal Processor
PMI	Personal Movement Identification
ADC	Analogue to Digital Converter
